# A radiomics-boosted deep-learning for risk assessment of synchronous peritoneal metastasis in colorectal cancer

**DOI:** 10.1186/s13244-024-01733-5

**Published:** 2024-06-18

**Authors:** Ding Zhang, BingShu Zheng, LiuWei Xu, YiCong Wu, Chen Shen, ShanLei Bao, ZhongHua Tan, ChunFeng Sun

**Affiliations:** 1 Medical School of Nantong University, Nantong, JiangSu China; 2 Department of Nuclear Medicine, Affiliated Hospital of Nantong University, Nantong, JiangSu China; 3 Department of General Surgery, Affiliated Hospital of Nantong University, Nantong, JiangSu China

**Keywords:** Peritoneal metastasis, Colorectal cancer, Radiomics, Deep learning, 18F-FDG-PET/CT

## Abstract

**Objectives:**

Synchronous colorectal cancer peritoneal metastasis (CRPM) has a poor prognosis. This study aimed to create a radiomics-boosted deep learning model by PET/CT image for risk assessment of synchronous CRPM.

**Methods:**

A total of 220 colorectal cancer (CRC) cases were enrolled in this study. We mapped the feature maps (Radiomic feature maps (RFMs)) of radiomic features across CT and PET image patches by a 2D sliding kernel. Based on ResNet50, a radiomics-boosted deep learning model was trained using PET/CT image patches and RFMs. Besides that, we explored whether the peritumoral region contributes to the assessment of CRPM. In this study, the performance of each model was evaluated by the area under the curves (AUC).

**Results:**

The AUCs of the radiomics-boosted deep learning model in the training, internal, external, and all validation datasets were 0.926 (95% confidence interval (CI): 0.874–0.978), 0.897 (95% CI: 0.801–0.994), 0.885 (95% CI: 0.795–0.975), and 0.889 (95% CI: 0.823–0.954), respectively. This model exhibited consistency in the calibration curve, the Delong test and IDI identified it as the most predictive model.

**Conclusions:**

The radiomics-boosted deep learning model showed superior estimated performance in preoperative prediction of synchronous CRPM from pre-treatment PET/CT, offering potential assistance in the development of more personalized treatment methods and follow-up plans.

**Critical relevance statement:**

The onset of synchronous colorectal CRPM is insidious, and using a radiomics-boosted deep learning model to assess the risk of CRPM before treatment can help make personalized clinical treatment decisions or choose more sensitive follow-up plans.

**Key Points:**

- Prognosis for patients with CRPM is bleak, and early detection poses challenges.
- The synergy between radiomics and deep learning proves advantageous in evaluating CRPM.
- The radiomics-boosted deep-learning model proves valuable in tailoring treatment approaches for CRC patients.

**Graphical Abstract:**

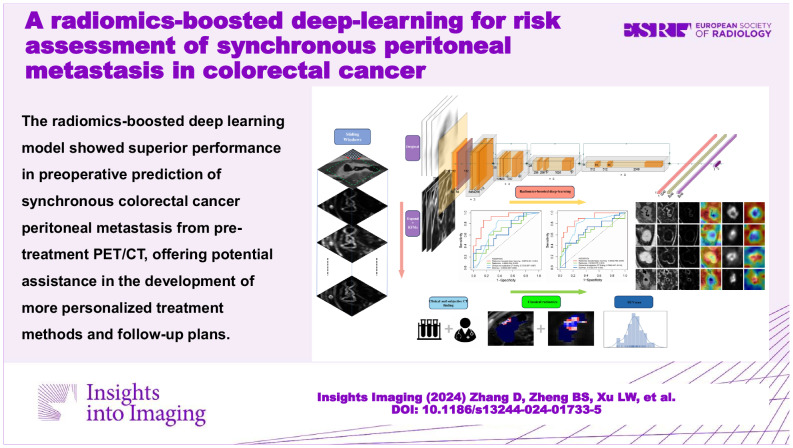

## Introduction

Colorectal cancer (CRC) ranks as the second leading cause of cancer-related deaths [1]. At the time of initial diagnosis, 5–15% of CRC patients present with peritoneal metastases [2]. Synchronous colorectal cancer peritoneal metastasis (CRPM) refers to cases where peritoneal metastasis (PM) occurs either at the time of initial diagnosis or within six months after surgery [3]. The prognosis for PM is grim, lacking standard treatment, and average life expectancy ranges from 6 to 12 months [4, 5]. Although a combination of bevacizumab and cetuximab can extend overall survival (OS) by 18.2 months for CRPM patients, its efficacy remains inferior to that for liver and lung metastases [6]. A multicenter phase III clinical trial demonstrated the potential of cytoreductive surgery (CRS) in treating CRPM, yielding a median OS of 41.2 months [7]. Accurate diagnosis of CRPM is crucial; however, the diagnostic process is challenging, and not all patients can undergo laparoscopic examination for pathological confirmation.

Noninvasive methods are commonly used to predict PM, among which serum tumor markers such as carcinoembryonic antigen (CEA) and carbohydrate antigen 19-9 (CA19-9) play an important role, which reflects to varying degrees of tumor invasion, proliferation, and invasion of peritoneal mesothelial cells, but not all patients exhibit elevated tumor markers, making them supplementary diagnostic tools [8, 9]. Computed tomography’s (CT) sensitivity to peritoneal lesions smaller than 0.5 cm is limited to 11–48% [10]. While diffusion-weighted magnetic resonance imaging (DW-MRI) and PET/CT are highly sensitive and specific for CRPM, they can be influenced by factors such as respiratory and intestinal motility, changes in lesion size, and pathological types. Particularly, 18F-fluorodeoxyglucose PET/CT (18F-FDG PET/CT) is valuable for diagnosing and staging various malignancies, including CRC, but distinguishing peritoneal cancer lesions is challenging due to physiological FDG uptake in the normal gut and lower uptake in certain mucinous tumors [11, 12]. Additionally, inter-reader variability in imaging diagnosis cannot be entirely avoided.

Radiomics and deep learning algorithms are gaining recognition in medical imaging analysis [13, 14]. Extracting handcrafted radiomic features allows for sensitive detection of subtle heterogeneity in tumor morphology or function. Li et al demonstrated high accuracy in predicting CRPM status by utilizing CT texture extracted from primary tumor lesions and the largest metastatic lymph node [14]. However, handcrafted radiomic features heavily rely on accurately delineating lesion boundaries. In contrast, convolutional neural networks, by directly learning specific features from input images, enhance accuracy and eliminate the need for precise lesion depiction [15].

This study leverages the complementary nature of handcrafted radiomics and deep learning to develop a radiomics-boosted deep learning model for preoperative risk assessment of synchronous CRPM based on PET/CT images. Notably, there are no reports on the application of PET/CT-based radiomics and deep learning methods for predicting synchronous CRPM.

## Methods and materials

### Patient selection

**Fig. 1:**
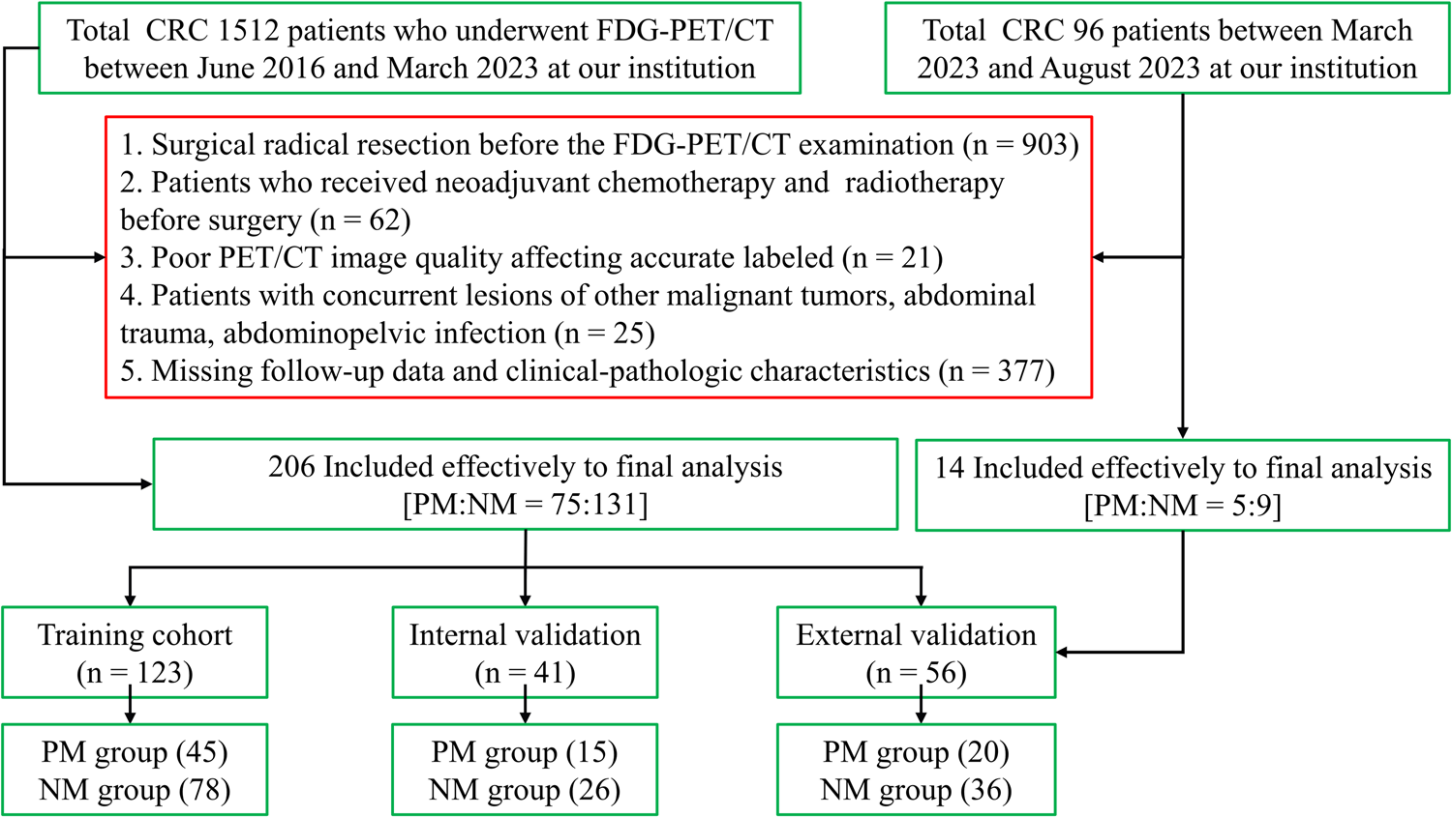
Participant recruitment flowchart for the study

This retrospective study received approval from the Ethics Committee of the Affiliated Hospital of Nantong University, and informed consent was waived due to its retrospective nature. The study included 220 patients diagnosed with CRC at Nantong University Affiliated Hospital between June 2016 and August 2023. Prior to March 2023, patients were randomly divided into training (*n* = 123), internal validation (*n* = 41), and external validation cohorts at a 6:2:2 ratio. Subsequently, all patients diagnosed from March 2023 to August 2023 were assigned to external validation cohorts (*n* = 56). Eighty with PM and 140 non-metastasis (NM) were included. Inclusion criteria comprised: (1) undergoing FDG-PET/CT scans before any treatment; (2) confirming CRC diagnosis through surgery or biopsy; (3) availability of follow-up data and clinical-pathologic information. Exclusion criteria included: (1) undergoing neoadjuvant chemotherapy and radiotherapy before surgery; (2) poor PET/CT image quality affecting accurate labeling; (3) abdominal trauma, abdominopelvic infection, or concurrent lesions of other malignant tumors. Detailed cohort inclusion is presented in Fig. 1. Clinicopathological data and lab results were collected within two weeks before the PET/CT scan. Details of synchronous CRPM status evaluation are provided in Supplementary A1.

### Region of interest (ROI) patch generation

**Fig. 2:**
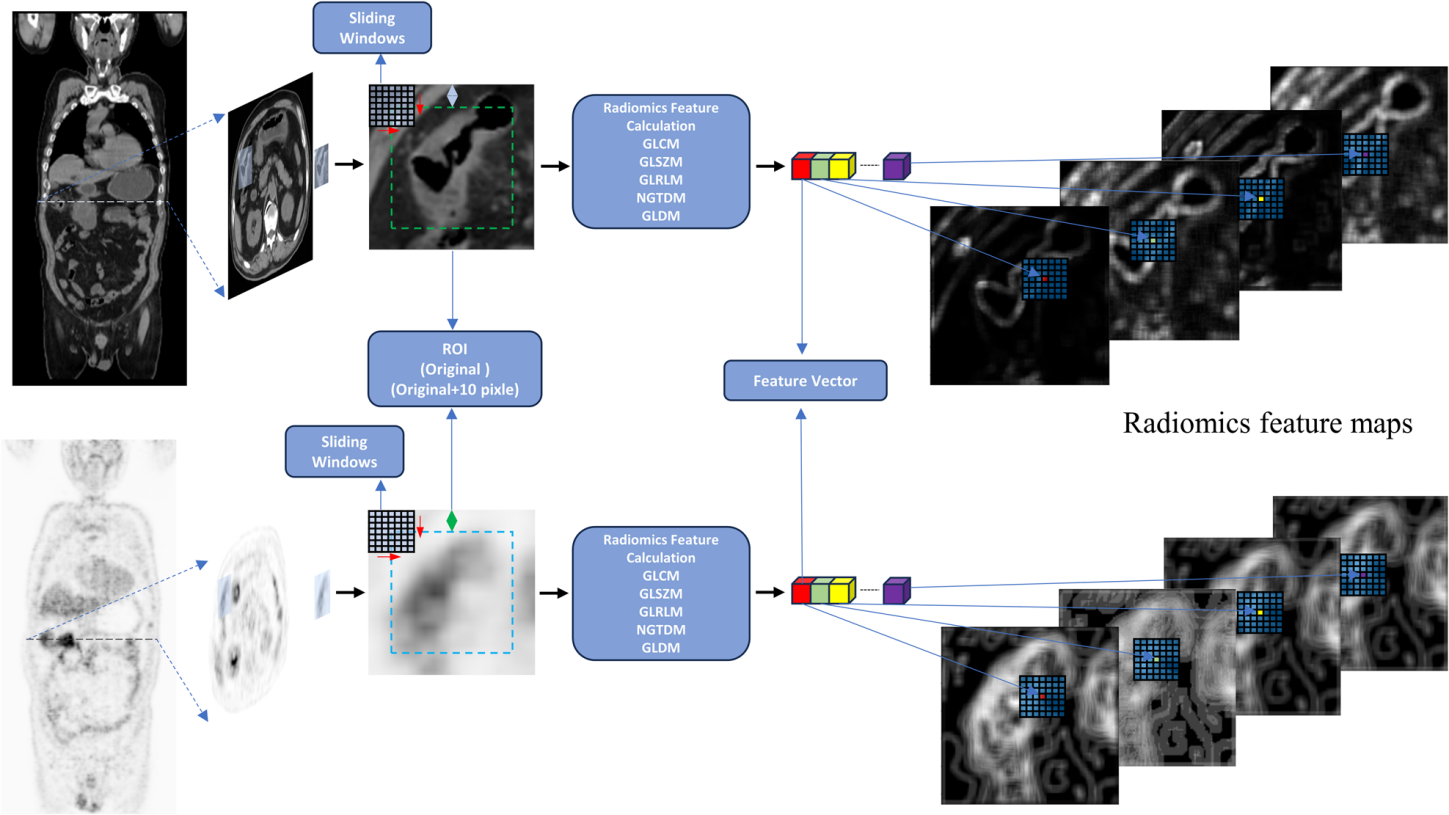
Workflow of RFM calculation in the study. RFM, radiomics feature map

Details of 18F-fluorodeoxyglucose positron emission tomography/computed tomography (18F-FDG-PET/CT) examinations, image preprocessing, and subjective CT finding evaluation are outlined in Supplementary A2. Using Lifex software (version 7.23) [16], original regions of interest (ROIs) were drawn along the lesion contour on the largest tumor cross-sectional image section in the axial direction. A minimum rectangle boundary was created around each manual original ROI, and cropping was performed to obtain original ROI patches. To explore the potential contributions of surrounding tissue, we expanded 10 pixels around the minimum bounding box of the ROI, creating expanded ROI patches (Fig. 2). All images were resized to 224 × 224.

### Radiomic feature maps (RFMs) generation

Classical radiomics is commonly utilized to capture the overall texture of ROIs, but it may not effectively discern subtle texture variations. To address this limitation, we devised a workflow for computing RFMs, as illustrated in Fig. 2. Utilizing a 7 × 7 matrix-size kernel, we extracted 75 radiomic features from the ROI, specifically including GLCM × 24, GLDM × 14, GLRLM × 16, GLSZM × 16, and NGTDM × 5 (refer to Table 1) [17]. Employing the kernel, we generated 75 feature maps with the same dimensions as the ROI patch by sliding it across the ROI patches, with the center value of the kernel being filled into each pixel.

**Table 1:** Seventy-five radiomics features included in this study

GLCM-based features	1. Autocorrelation	GLRLM-based features	39. GrayLevelNonUniformity
	2. ClusterProminence		40. GrayLevelNonUniformityNormalized
	3. ClusterShade		41. GrayLevelVariance
	4. ClusterTendency		42. HighGrayLevelRunEmphasis
	5. Contrast		43. LongRunEmphasis
	6. Correlation		44. LongRunHighGrayLevelEmphasis
	7. DifferenceAverage		45. LongRunLowGrayLevelEmphasis
	8. DifferenceEntropy		46. LowGrayLevelRunEmphasis
	9. DifferenceVariance		47. RunEntropy
	10. Id		48. RunLengthNonUniformity
	11. Idm		49. RunLengthNonUniformityNormalized
	12. Idmn		50. RunPercentage
	13. Idn		51. RunVariance
	14. Imc1		52. ShortRunEmphasis
	15. Imc2		53. ShortRunHighGrayLevelEmphasis
	16. InverseVariance		54. ShortRunLowGrayLevelEmphasis
	17. JointAverage	GLSZM-based features	55. GrayLevelNonUniformity
	18. JointEnergy		56. GrayLevelNonUniformityNormalized
	19. JointEntropy		57. GrayLevelVariance
	20. MCC		58. HighGrayLevelZoneEmphasis
	21. MaximumProbability		59. LargeAreaEmphasis
	22. SumAverage		60. LargeAreaHighGrayLevelEmphasis
	23. SumEntropy		61. LargeAreaLowGrayLevelEmphasis
	24. SumSquares		62. LowGrayLevelZoneEmphasis
GLDM-based features	25. DependenceEntropy		63. SizeZoneNonUniformity
	26. DependenceNonUniformity		64. SizeZoneNonUniformityNormalized
	27. DependenceNonUniformityNormalized		65. SmallAreaEmphasis
	28. DependenceVariance		66. SmallAreaHighGrayLevelEmphasis
	29. GrayLevelNonUniformity		67. SmallAreaLowGrayLevelEmphasis
	30. GrayLevelVariance		68. ZoneEntropy
	31. HighGrayLevelEmphasis		69. ZonePercentage
	32. LargeDependenceEmphasis		70. ZoneVariance
	33. LargeDependenceHighGrayLevelEmphasis	NGTDM-based features	71. Busyness
	34. LargeDependenceLowGrayLevelEmphasis		72. Coarseness
	35. LowGrayLevelEmphasis		73. Complexity
	36. SmallDependenceEmphasis		74. Contrast
	37. SmallDependenceHighGrayLevelEmphasis		75. Strength
	38. SmallDependenceLowGrayLevelEmphasis		

For each feature, the feature value was assigned by centering at the ROI, utilizing a moving kernel across the ROI patch as a sliding window operation. This process resulted in the formation of 75 feature maps, maintaining the same dimensions as the original ROI patches.

To mitigate feature redundancy, for each patient in the training cohort, we calculated Pearson correlation coefficients (*r*) for each pair of RFMs, leading to the creation of 123 covariance matrices. Highly correlated feature maps with *r* > 0.95 were then excluded in the subsequent average Pearson covariance matrix [18].

### Neural network architecture

**Fig. 3:**
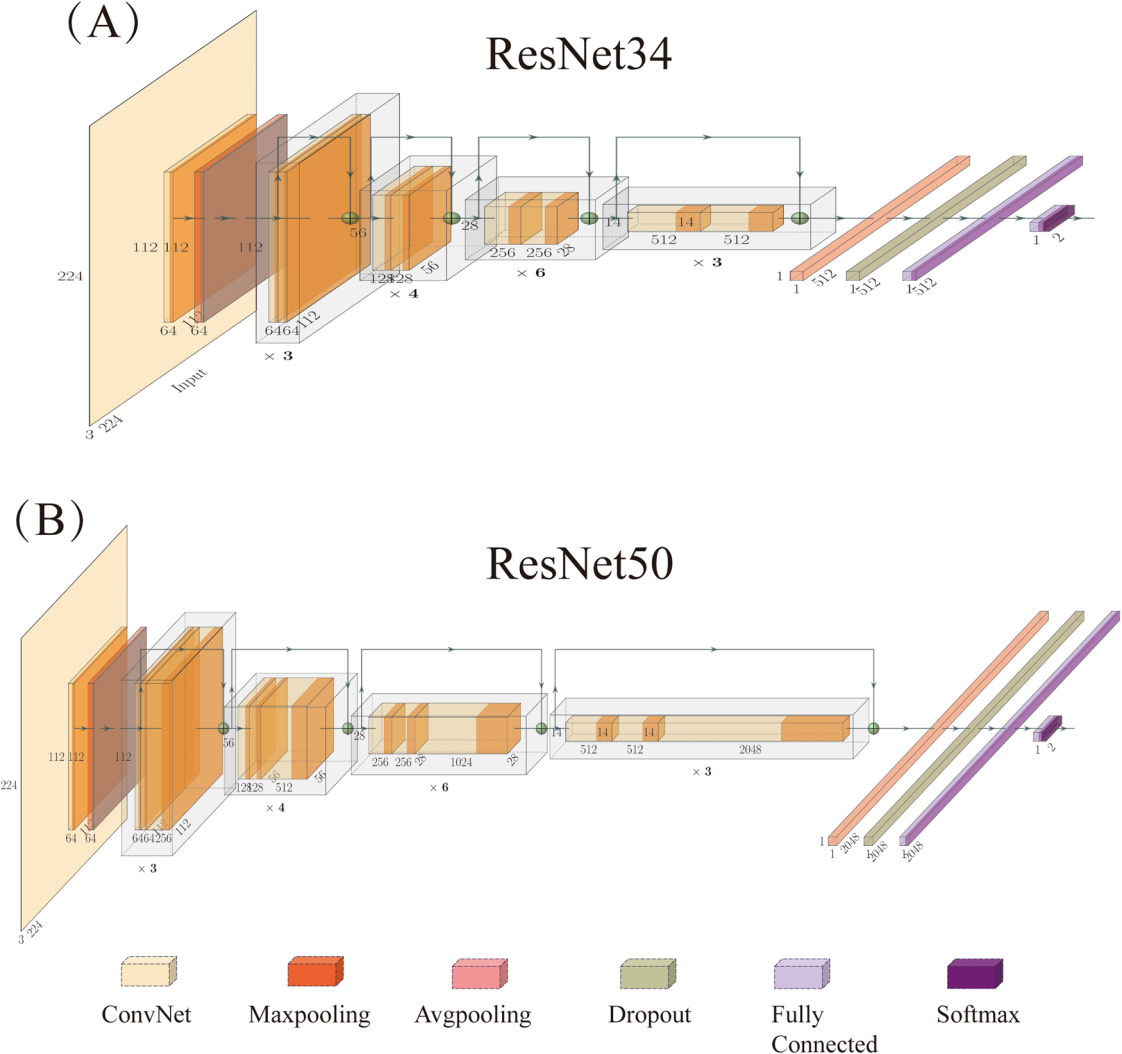
Two deep neural networks studied. **A** ResNet34. **B** ResNet50

We explored two neural network architectures, namely ResNet34 (Fig. 3A) and ResNet50 (Fig. 3B). To tailor the models to the specific problem, we employed transfer learning and fine-tuning techniques, including: (1) initializing the convolutional bases with pre-trained weights from ImageNet and (2) treating the last two fully connected layers (FC) as free parameters for training specific tasks [19]. To prevent overfitting, a dropout was placed between the two FC layers, and the output utilized softmax activation. Detailed parameters of the deep learning model can be found in Supplementary A3.

For the pilot model, the sole input comprised original and expanded ROI patches. Grayscale ROI patches were broadcast to three channels to align with pre-trained neural network input shapes. In the radiomics-boosted model, a three-channel image was generated by stacking grayscale ROI patches and two RFMs as input variables. The two RFMs were selected based on the saliency map (SM) of the pilot model, indicating the importance of each pixel for the final classification. Pixels with higher intensity values in the SM were deemed more critical for neural networks to diagnose, so two RFMs were chosen whose average correlation with SM was the highest in the training dataset. For each model, we assessed stability through 50 runs. Subsequently, the model with the best average area under the curve (AUC) and its input variables were selected as the final model. The model closest to the average AUC was chosen to obtain output probabilities for all cases. Finally, a PET/CT radiomics-boosted deep-learning model based on network output probability was constructed using multivariable logistic analysis.

### Model comparison

#### Radiomics-boosted deep-learning compared with classical radiomics model

We conducted a comparative analysis between the radiomics-boosted deep-learning model and the classical radiomics model to assess their effectiveness. The construction process of the classical radiomics model is detailed in Supplementary A4.

#### Radiomics-boosted deep-learning compared with clinical and subjective CT finding model

Univariate logistic regression analysis was employed to investigate the impact of clinical information and subjective CT findings on CRPM risk. The clinical and subjective CT finding model was developed using multivariable logistic analysis with backward stepwise selection.

#### Maximum standardized uptake value (SUVmax) based classification

SUVmax is a widely recognized semiquantitative parameter in PET/CT studies. We evaluated a logistic regression model based on SUVmax as a baseline comparison in our cohorts [20, 21].

### Statistical analyses

R version 4.2.2 (http://www.r-project.org) was utilized for statistical analyses. Categorical variables were presented as counts with proportions, while continuous variables with normally distributed and non-normally distributed data were expressed as mean ± SD, median, and interquartile range, respectively. A significance level of *p* < 0.05 on both sides was considered statistically significant. ROC analyses were conducted using the “pROC” package. Wilcoxon signed rank tests were employed to compare results from 50 runs in the pilot model.

## Results

### Clinical information and subjective CT findings

Table 2 displays the demographics and subjective CT findings across all cohorts. Patients were categorized into two groups based on follow-up results: PM and NM. Significant differences were observed in tumor infiltration into the surrounding fat and the level of CA19-9 between the two groups across all three cohorts. Primary tumor location, SUVmax, and levels of CEA exhibited differences in the training and external validation cohorts. Patient age reached statistical significance only in the internal validation cohort (*p* < 0.05).

**Table 2:** Demographic and subjective CT characteristics in the three cohorts

	Total	Training, (*n* = 123)			Internal validation, (*n* = 41)			External validation, (*n* = 56)		
	*N* = 220	NM, (*n* = 78)	PM, (*n* = 45)	*p*	NM, (*n* = 26)	PM, (*n* = 15)	*p*	NM, (*n* = 36)	PM, (*n* = 20)	*p*
Location										
Right	58 (26.4)	16 (20.5)	18 (40.0)	0.020^a^	6 (23.1)	5 (33.3)	0.475	5 (13.9)	8 (40.0)	0.027^a^
Left	162 (73.6)	62 (79.5)	27 (60.0)		20 (76.9)	10 (66.7)		31 (86.1)	12 (60.0)	
Gender										
Female	80 (36.4)	32 (41.0)	13 (28.9)	0.178	5 (19.2)	7 (46.7)	0.063	16 (44.4)	7 (35.0)	0.491
Male	140 (63.6)	46 (59.0)	32 (71.1)		21 (80.8)	8 (53.3)		20 (55.6)	13 (65.0)	
Age										
Median (IQR]/mean (± SD)	68.0 (59.0, 76.0)	66.0 (59.0, 73.0)	66.0 (55.0, 78.0)	0.920	71.1 ± 11.6	61.3 ± 15.3	0.031^a^	71.0 (62.0, 77.0)	67.0 (62.0, 76.0)	0.700
Weight, mean (± SD)	63.6 ± 11.2	63.7 ± 10.8	62.7 ± 12.9	0.650	65.0 ± 10.7	62.2 ± 8.4	0.928	64.6 ± 10.9	63.2 ± 10.6	0.641
BMI, mean (± SD)	23.1 ± 3.4	23.3 ± 3.3	22.5 ± 3.9	0.214	23.5 ± 3.3	22.5 ± 2.6	0.363	23.3 ± 2.7	23.3 ± 3.2	0.965
SUVmax										
Median (IQR)	17.4 (13.5, 23.5)	18.7 (14.8, 24.5)	15.4 (10.7, 21.3)	0.006^a^	16.0 (14.6, 25.7)	15.2 (10.9, 16.9)	0.060	20.3 (13.8, 23.0)	14.0 (10.4, 18.7)	0.035^a^
Thickness, median (IQR)	1.4 (1.1, 1.8)	1.3 (1.1, 1.8)	1.5 (1.0, 2.0)	0.408	1.4 (1.2, 1.8)	1.3 (1.2, 1.6)	0.350	1.3 (1.1, 1.7)	1.5 (1.2, 2.0)	0.355
CEA										
(−, ≤ 5 ng/mL)	99 (45.0)	42 (54.5)	11 (23.9)	0.016^a^	15 (57.7)	6 (40.0)	0.275	20 (55.6)	5 (25.0)	0.028^a^
(+, > 5 ng/mL)	121 (55.0)	35 (45.5)	35 (76.1)		11 (42.3)	9 (60.0)		16 (44.4)	15 (75.0)	
CA19-9										
(−, ≤ 37 U/mL)	135 (61.4)	65 (83.3)	19 (42.2)	< 0.001^a^	15 (57.7)	3 (20.0)	0.019^a^	25 (69.4)	8 (40.0)	0.032^a^
(+, > 37 U/mL)	85 (38.6)	13 (16.7)	26 (57.8)		11 (42.3)	12 (80.0)		11 (30.6)	12 (60.0)	
Infiltration										
Absent	124 (56.4)	49 (62.8)	19 (42.2)	0.027^a^	19 (73.1)	6 (40.0)	0.036^a^	24 (66.7)	7 (35.0)	0.022^a^
Present	96 (43.6)	29 (37.2)	26 (57.8)		7 (26.9)	9 (60.0)		12 (33.3)	13 (65.0)	

*CEA* carcinoembryonic antigen, *CA19-9* carbohydrate antigen 19-9

^a^ Indicating statistical significance

### Predictive performance of the pilot model

Table 3 provides a summary of quantitative comparisons, including sensitivity, specificity, accuracy, positive predictive value (PPV), negative predictive value (NPV), AUC, and average AUC of 50 runs, comparing the pilot models and radiomics-boosted models in both the training and internal validation cohorts. In all pilot models, using expanded ROI patches as the input variable achieved higher average AUC values in the validation cohort (*p* < 0.001). Comparing ResNet34 and ResNet50 models, the latter achieved average AUC gains with any CT patches and used PET expand ROI patches as input variables, yielding similar results (*p* < 0.001) (Fig. S1 A, B, D, and E). Ultimately, two optimal models were selected from the pilot models: ResNet50 with CT and PET expand ROI patches as input variables, exhibiting higher AUC and lower standard deviation (SD), indicating a high level of robustness (0.739 ± 0.027 and 0.798 ± 0.007, respectively). The AUC, accuracy, sensitivity, specificity, PPV, and NPV of the CT model that is closest to the average AUC value in the internal validation cohort are 0.749, 0.659, 0.600, 0.692, 0.529, and 0.750, respectively, while in the PET model, they are 0.797, 0.732, 0.933, 0.615, 0.583, and 0.941, respectively.

**Table 3:** Performance of pilot models with different input variables and different neural networks

Mode	Modality	Patches	Training cohort						Validation cohort						
			AUC	ACC	SEN	SPE	PPV	NPV	AUC	ACC	SEN	SPE	PPV	NPV	AUC (50 repeats)
ResNet34	CT	Original	0.613	0.545	0.711	0.449	0.427	0.729	0.513	0.512	0.800	0.346	0.414	0.750	0.514 ± 0.064
		Expand	0.775	0.691	0.844	0.603	0.551	0.870	0.656	0.583	0.5333	0.615	0.444	0.696	0.656 ± 0.039
	PET	Original	0.761	0.780	0.578	0.897	0.787	0.765	0.654	0.658	0.600	0.692	0.750	0.529	0.655 ± 0.033
		Expand	0.833	0.748	0.778	0.731	0.625	0.851	0.718	0.658	0.667	0.654	0.526	0.772	0.725 ± 0.019
ResNet50	CT	Original	0.797	0.748	0.778	0.731	0.452	0.851	0.667	0.667	0.538	0.538	0.455	0.737	0.679 ± 0.029
		Expand	0.787	0.844	0.551	0.658	0.521	0.860	0.749	0.659	0.600	0.692	0.529	0.750	0.739 ± 0.027
	PET	Original	0.781	0.699	0.578	0.769	0.591	0.759	0.651	0.610	0.600	0.515	0.473	0.727	0.636 ± 0.023
		Expand	0.819	0.707	0.733	0.692	0.579	0.818	0.797	0.732	0.933	0.615	0.583	0.941	0.798 ± 0.007
ResNet50	CT	Expand + RFM	0.884	0.740	0.889	0.654	0.597	0.911	0.869	0.780	0.800	0.769	0.667	0.870	0.866 ± 0.018
	PET	Expand + RFM	0.809	0.756	0.444	0.936	0.810	0.725	0.782	0.415	1.000	0.115	0.395	1.000	0.796 ± 0.008

*RFM* radiomic feature maps

### RFM selection and radiomics-boosted deep-learning model construction

**Fig. 4:**
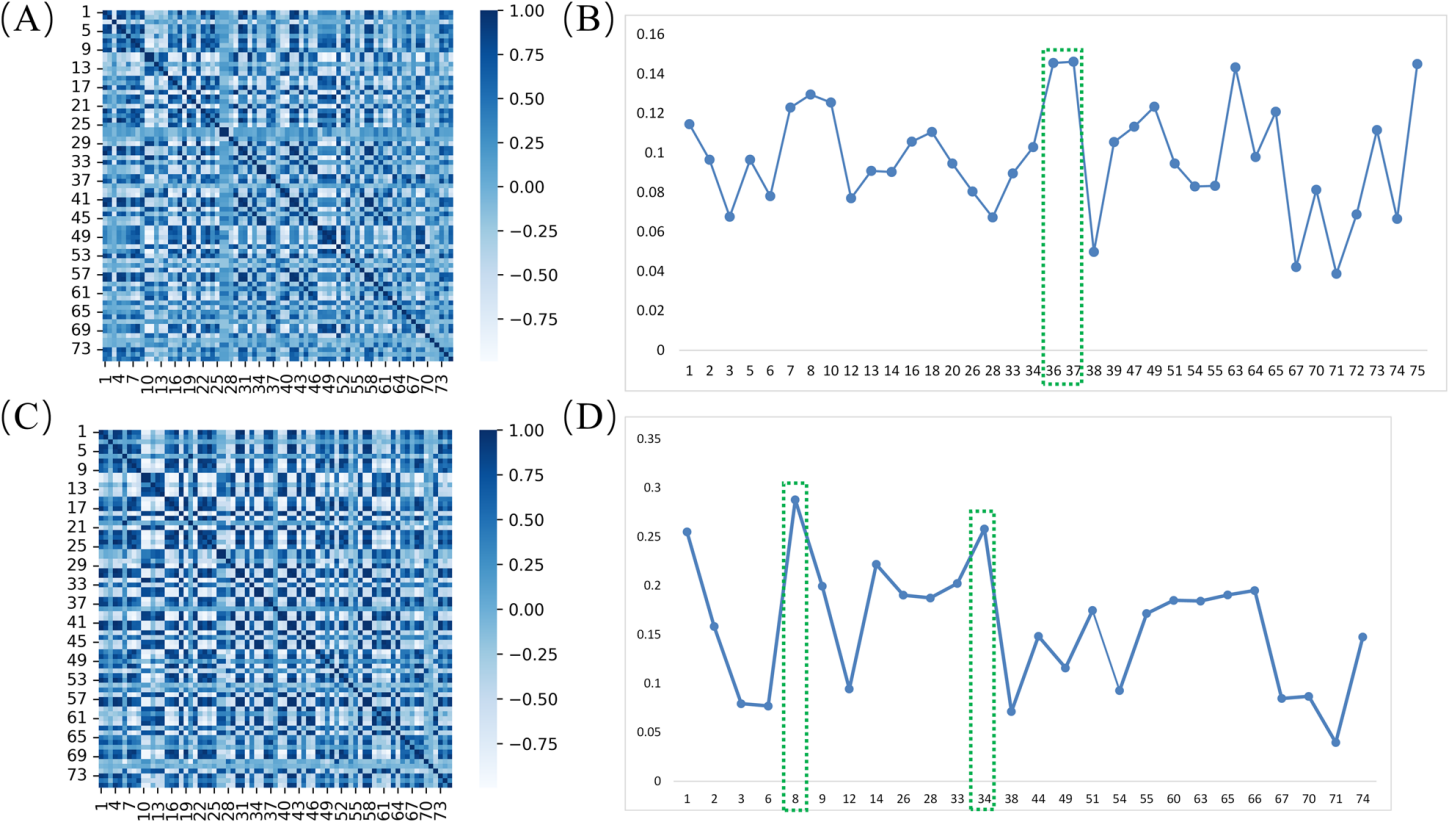
RFM selection. Average correlation heatmap of RFM generated by expanded ROI patches from CT (**A**) and PET (**C**) images. Panels **B**, **D** depict the average correlation values between RFM and SM of CT and PET pilot models after removing redundant features, highlighting the highest-performing features with green boxes. RFM, radiomics feature map; SM, saliency map

Figure 4A, C illustrates the average correlation heatmap of RFM generated by expanded ROI patches from CT and PET images. After excluding highly correlated features, the RFMs of CT and PET include 37 and 27 features. The average correlation values between RFMs and the SM from the two pilot models are depicted in Fig. 4B, D, highlighting the highest-performing features with green boxes. For the RFMs of CT patches, GLDM-based small dependence emphasis (SDE) (0.145) and GLDM-based small dependence high gray level emphasis (SDHGLE) (0.146) achieved the highest average correlation. Similarly, for the RFMs of PET patches, GLCM-based difference entropy (DE) (0.288) and GLDM-based large dependence low gray level emphasis (LDLGLE) (0.258) were selected. The overall average correlation value of RFMs generated from CT is lower than PET.

Compared to the ResNet50 using only CT expand ROI patches as input, the model’s performance significantly improved after adding RFMs (AUC from 0.739 ± 0.027 to 0.866 ± 0.018, *p* < 0.001) (Fig. S1 C). The AUC, accuracy, sensitivity, specificity, PPV, and NPV in the internal validation cohort are 0.869, 0.780, 0.800, 0.769, 0.667, and 0.870. Unexpectedly, the performance of the radiomics-boosted model decreased in PET (AUC from 0.798 ± 0.007 to 0.796 ± 0.008, *p* = 0.006) (Fig. S1 F and Table 3).

**Fig. 5:**
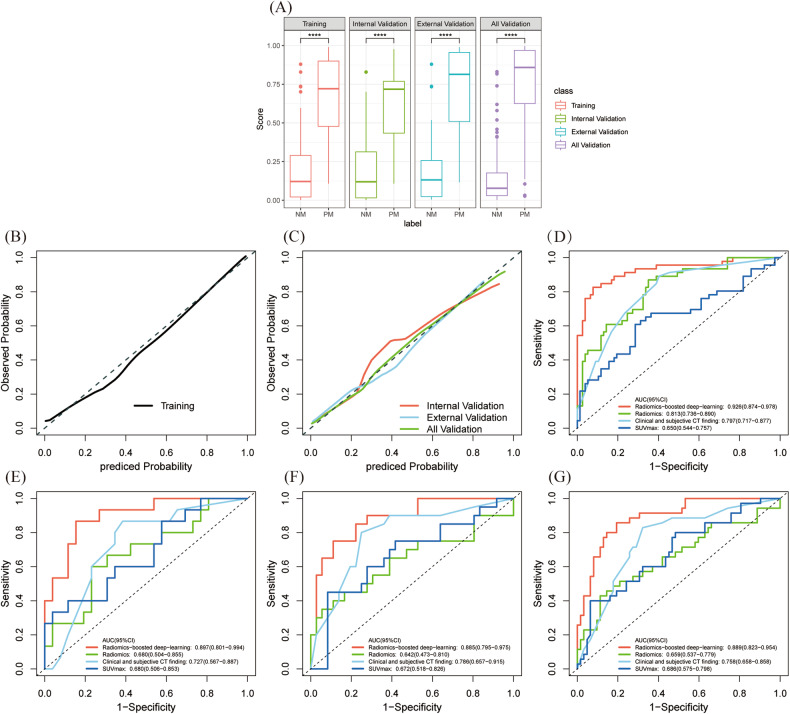
Performance evaluation of prediction models. Distribution of the radiomics-boosted deep-learning model score in all datasets (**A**). Calibration curves of the radiomics-boosted deep-learning model in training (**B**) and validation cohorts (**C**). Panels **D**–**G** show AUCs of different models in all training, internal, external, and all validation datasets. AUC, area under the curves

After a comprehensive comparison of various models, two best models based on PET/CT images were identified: (1) ResNet50 with CT ROI expand patches + RFMs as input. (2) ResNet50 with only PET ROI expand patches as input. The radiomics-boosted deep-learning model score was obtained by multivariable logistic regression on the output probabilities of the two models. This model not only exhibits the best predictive performance in the training and internal validation cohorts but also has high generalization ability in the external validation cohort. The AUC, accuracy, sensitivity, specificity, PPV, and NPV in the internal validation cohort are 0.897 (95% confidence interval (CI): 0.801–0.994), 0.829, 0.800, 0.846, 0.750, and 0.880, respectively, 0.885 (95% CI: 0.795–0.975), 0.821, 0.700, 0.889, 0.778, and 0.842 in the external validation cohort. In all validation datasets, these values were 0.889 (95% CI: 0.823–0.954), 0.825, 0.743, 0.871, 0765, and 0.857 (Table 4). All datasets showed differences between the NM and PM groups (Fig. 5A). According to the Hosmer-Lemeshow test, the ideal curve and the predictive calibration curve are similar in both training, internal, external, and all validation datasets (*p* = 0.225, 0.224, 0.447, 0.696, respectively) (Fig. 5B, C).

**Table 4:** Comparison of the prediction performance of four models for synchronous CRPM risk

Data set	Model	AUC (95% CI)	Accuracy	Sensitivity	Specificity	PPV	NPV
Training cohort, (*n* = 123)	Radiomics-boosted deep-learning model	0.926 (0.874–0.978)	0.886 (109/123)	0.826 (38/46)	0.922 (71/77)	0.864 (38/44)	0.899 (71/79)
	Clinical and subjective CT finding	0.797 (0.717–0.877)	0.707 (87/123)	0.891 (41/46)	0.597 (46/77)	0.569 (41/72)	0.902 (46/51)
	Radiomics	0.813 (0.736–0.890)	0.732 (90/123)	0.870 (40/46)	0.650 (50/77)	0.597 (40/67)	0.893 (50/56)
	SUVmax	0.650 (0.544–0.757)	0.675 (83/123)	0.609 (28/46)	0.714 (55/77)	0.560 (22/50)	0.753 (55/73)
Internal validation cohort, (*n* = 41)	Radiomics-boosted deep-learning model	0.897 (0.801–0.994)	0.829 (34/41)	0.800 (12/15)	0.846 (22/26)	0.750 (12/16)	0.880 (22/25)
	Clinical and subjective CT finding	0.727 (0.567–0.887)	0.634 (26/41)	0.867 (13/15)	0.500 (13/26)	0.500 (13/26)	0.867 (13/15)
	Radiomics	0.680 (0.504–0.855)	0.585 (24/41)	0.733 (11/15)	0.500 (13/26)	0.458 (11/24)	0.765 (13/17)
	SUVmax	0.680 (0.506–0.853)	0.561 (23/41)	0.600 (9/15)	0.539 (14/26)	0.429 (9/21)	0.700 (14/20)
External validation cohort, (*n* = 56)	Radiomics-boosted deep-learning model	0.885 (0.795–0.975)	0.821 (46/56)	0.700 (14/20)	0.889 (32/36)	0.778 (14/18)	0.842 (32/38)
	Clinical and subjective CT finding	0.786 (0.657–0.915)	0.714 (40/56)	0.850 (17/20)	0.639 (23/36)	0.567 (17/30)	0.885 (23/26)
	Radiomics	0.642 (0.473–0.810)	0.643 (36/56)	0.450 (9/20)	0.750 (27/36)	0.500 (9/18)	0.711 (27/38)
	SUVmax	0.672 (0.518–0.826)	0.643 (36/56)	0.600 (12/20)	0.667 (24/36)	0.500 (12/24)	0.750 (24/32)
All validation cohorts, (*n* = 97)	Radiomics-boosted deep-learning model	0.889 (0.823–0.954)	0.825 (80/97)	0.743 (26/35)	0.871 (54/62)	0.765 (26/34)	0.857 (54/63)
	Clinical and subjective CT finding	0.758 (0.658–0.858)	0.680 (66/97)	0.857 (30/35)	0.581 (36/62)	0.536 (30/56)	0.878 (36/41)
	Radiomics	0.659 (0.537–0.779)	0.619 (60/97)	0.571 (20/35)	0.645 (40/62)	0.476 (20/42)	0.727 (40/55)
	SUVmax	0.686 (0.575–0.798)	0.598 (58/97)	0.600 (21/35)	0.597 (37/62)	0.457 (21/36)	0.725 (37/51)

### Model comparison

#### Radiomics-boosted deep-learning compared with classical radiomics model

The AUCs of the radiomics model in all cohorts are illustrated in Fig. S2 A–F. The PET/CT classical radiomics model score, calculated by multivariable logistic regression, yielded AUCs of 0.813 (95% CI: 0.736–0.890), 0.680 (95% CI: 0.504–0.855), 0.642 (95% CI: 0.473–0.810), and 0.659 (95% CI: 0.537–0.779) in the training, internal, external, and all validation datasets, respectively. The IDI and DeLong test demonstrated that the radiomics-boosted deep-learning model improved performance compared to the classical radiomics model (IDI = 0.302, *p* < 0.001; DeLong test, *p* < 0.001) (Fig. 5D–G).

#### Radiomics-boosted deep-learning compared with clinical and subjective CT finding model

Three clinical information and one subjective CT feature were screened using univariate and multivariate logistic regression to construct a clinical and subjective CT finding model (Table 5). The AUCs in the training, internal, external, and all validation datasets were 0.797 (95% CI: 0.717–0.877), 0.727 (95% CI: 0.567–0.887), 0.786 (95% CI: 0.657–0.915), and 0.758 (95% CI: 0.658–0.858), respectively. The performance was still lower than the radiomics-boosted deep-learning model (IDI = 0.206, *p* = 0.005; DeLong test, *p* = 0.024) (Fig. 5D–G).

**Table 5:** Univariate and multivariate logistic regression analysis for clinical information and subjective CT findings in the training cohort

Characteristics	Uni-variate analysis, (*p*)	Clinical and subjective CT finding model (*p*, AIC = 137.74)
Location		
Right		
Left	0.030^a^	0.020^a^
Gender		
Female		
Male	0.180	
Age	0.745	
Weight	0.647	
BMI	0.213	
Thickness	0.249	
CEA		
(−, ≤ 5 ng/mL)		
(+, > 5 ng/mL)	0.001^a^	0.046^a^
CA19-9		
(−, ≤ 37 U/mL)		
(+, > 37 U/mL)	< 0.001^a^	0.002^a^
Infiltration		
Absent		
Present	0.017^a^	0.043^a^

*CEA* carcinoembryonic antigen, *CA19-9* carbohydrate antigen 19-9

^a^ Indicating statistical significance

#### SUVmax assessment

The SUVmax showed limited performance, with AUCs of 0.650 (95% CI: 0.544–0.757) in the training cohort and 0.680 (95% CI: 0.506–0.853), 0.672 (95% CI: 0.518–0.826), and 0.686 (95% CI: 0.575–0.798) in the internal, external, and all validation datasets, respectively (Fig. 5D–G). Detailed data about AUC, accuracy, sensitivity, specificity, PPV, and NPV of all models are presented in Table 4.

## Discussion

This study utilized a new complementary approach of radiomics and deep learning for risk prediction of synchronous CRPM. The proposed radiomics-boosted deep learning model is completely superior to the clinical and subjective CT finding model, classical radiomics model, and SUVmax assessment, achieving optimal performance. We believe that for high-risk patients, it is necessary to search for metastatic lesions in more detail or develop a more compact and sensitive follow-up plan.

The independent predictive factors for CRPM in this study include elevated levels of CEA and CA19-9, which are consistent with previous research findings [22–25]. In addition, we also found that tumors situated in the right colon have a higher propensity for PM (*p* = 0.020). Previous research indicates that compared to adenocarcinomas in the left colon (32.5%), mucinous adenocarcinomas (42.3%) and signet ring cell carcinomas (48.8%) are more prevalent in the right colon, with respective probabilities of PM at 20.1%, 48.2%, and 51.2% (*p* < 0.001) [26]. Recent studies in CRC have explored the application of radiomics and deep learning techniques [27–31]. Li et al [14] developed a radionics-clinical fusion model based on texture features from primary tumor lesions and the largest metastatic lymph node, achieving good performance in predicting synchronous CRPM (training set AUC: 0.855, validation set: 0.793). Yuan et al [32] employed a ResNet3D + SVM-based deep learning framework, demonstrating potential in PM detection, albeit requiring substantial computational resources. Zhang et al [33] successfully developed a PM detection model with robust generalization using meta-learning algorithms, despite limited raw data availability, achieving an AUC of 0.728.

The onset of synchronous CRPM is insidious and symptoms lack specificity. National guidelines recommend the hematological, imaging examination, diagnostic laparoscopy, and cytological examination of abdominal fluid or perfusion fluid as primary diagnostic tools for CRPM [12, 34–36]. Nonetheless, due to the limitations of various methods, a significant number of patients are not diagnosed with PM until surgical exploration [37]. This study, involving a minimum six-month follow-up through pathology or imaging, developed classical radiomics and radiomics-boosted deep learning model based on pre-treatment PET/CT images. These models effectively assess the current risk and predict short-term significant progression of CRPM in patients, even in those initially assessed as negative. It can remind doctors to search for metastatic lesions in more detail or develop more sensitive follow-up plans. In this study, four CT and two PET radiomics features were constructed traditional radiomics models for synchronous CRPM risk evaluation (Supplementary A5). Notably, in our study, CT-derived features emphasized image texture complexity and uniformity, whereas PET features, exclusively first-order, depicted pixel or voxel distribution within the ROI, akin to physicians’ film interpretations, ignoring spatial correlations [17]. However, the classical radiomics model, reliant on predefined features, exhibits poor prediction accuracy (AUC: 0.659 (95% CI: 0.537–0.779) across all validation datasets). Previous research has highlighted synergies between deep learning and radiomics features, prompting this study to boost model efficacy through deep learning techniques, whilst leveraging the strengths of radiomics features [38–41].

To overcome the challenge of deep learning’s extensive training dataset requirement, we applied transfer learning and data augmentation to expand our dataset and prevent overfitting. The streamlined architecture and efficient training time of ResNet effectively captured predictive features, as demonstrated in our study and supported by other research [3, 42].

**Fig. 6:**
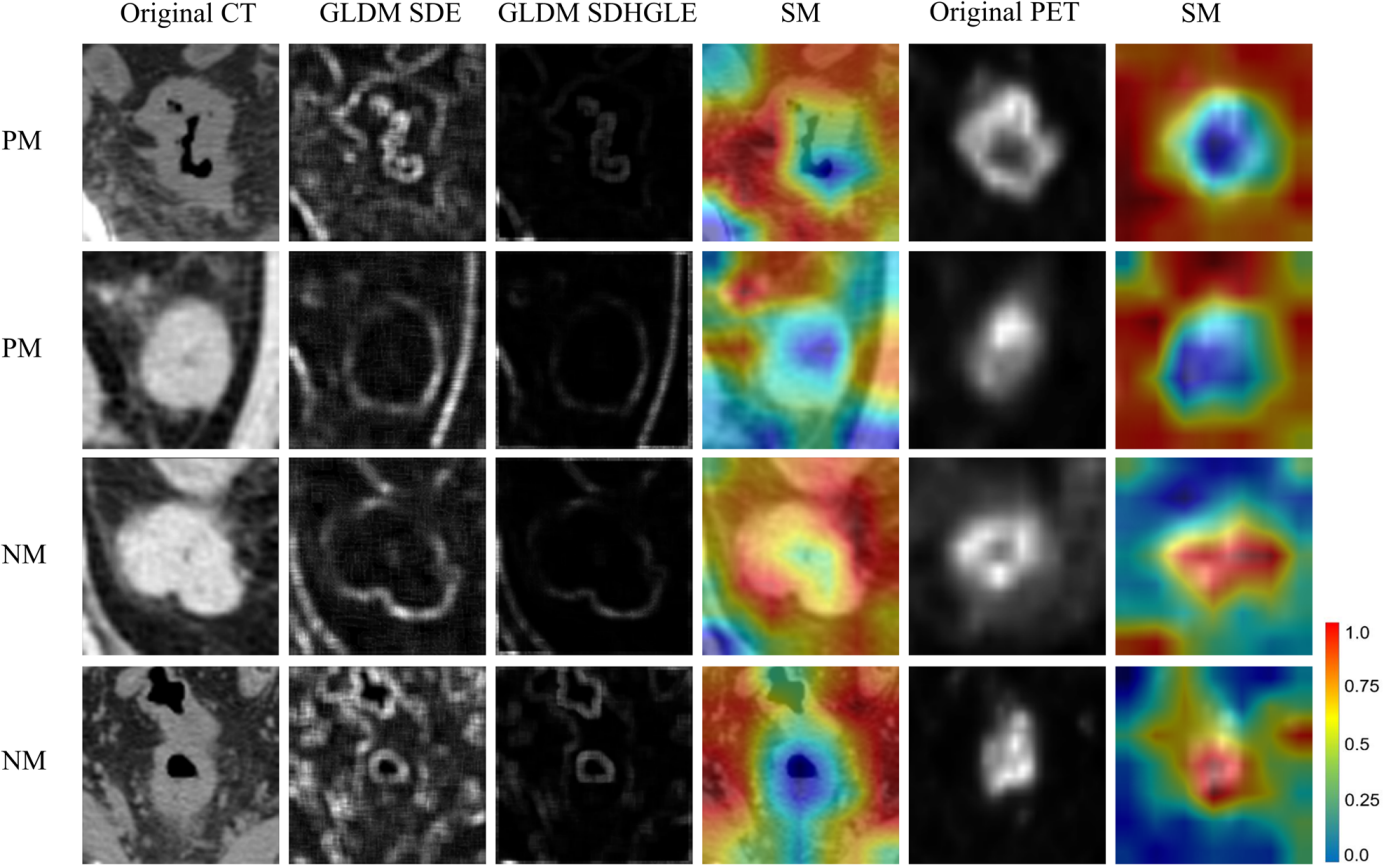
Radiomics-boosted deep learning model visualization. The first two rows depict patients with PM, and the last two rows of NM patients. The first and fifth columns are the original image patches of the PET/CT image. The surrounding area of the tumor in PM patients is relatively blurry on CT images. The second and third columns are the RFM generated based on the original CT slices, the relatively blurry tumor boundaries on CT patches are interrupted at the corresponding positions in RFM (GLDM SDE, GLDM SDHGLE). The fourth and sixth columns are visualizations of the radiomics-boosted deep learning model based on PET/CT images. The red color highlights the ROI. The CT model focuses on the surrounding tissues of the tumor, while the PET model focuses more on the distribution of SUVmax intensity. PM, peritoneal metastasis; NM, non-metastasis; RFM, radiomics feature map; GLDM SDE, GLDM-based small dependence emphasis; GLDM SDHGLE, GLDM-based small dependence high gray level emphasis

To comprehend the impact of tumor-surrounding tissues on classification, we annotated additional image patches encompassing the surrounding regions of the original ROI. The inclusion of surrounding tissue significantly enhanced the performance of both the CT and PET models, resulting in an average AUC of 0.739 ± 0.027 and 0.798 ± 0.007 in the validation cohort. This observation is consistent with the subjective assessment of tumor-surrounding fat infiltration on CT images (*p* < 0.05 in all cohorts). In CRC, the pT4 category, indicating serosal involvement by tumor cells, has been identified as a crucial independent prognostic factor, surpassing local spread and lymph node involvement [43]. Santvoort et al [44] advocate for diagnostic laparoscopy before selective resection in patients with radiological suspicion of T4 CRC. The introduction of RFMs is a noteworthy highlight, capturing subtle texture variations within ROIs driven by anatomical factors, as opposed to radiomic features calculated as scalar values. Prior studies on lung diseases have validated the rationality of this method, suggesting that the potential functional information in RFMs can enhance the accuracy of preoperative risk assessment for PM in CRC patients [18, 45]. The superimposition of CT images and RFMs yielded significant performance benefits, with GLDM-based SDE and GLDM-based SDHGLE having the highest correlation to SM. GLDM-based SDE assesses the distribution of small dependencies, with a greater value indicating smaller dependence and less homogeneous textures. GLDM-based SDHGLE measures the joint distribution of small dependence with higher gray-level values. The tumor boundary in two sets of RFMs is better defined than in the original image, and if fat infiltration is present around the tumor in the original image, the pixel value in the corresponding position in the RFMs decreases accordingly (Fig. 6).

The neural network model based on PET original ROI patches exhibits good predictive performance, but after radiomics enhancement, the performance is suppressed. This may be attributed to the rich tissue metabolism information in the original image, but a low number of pixels and the lack of anatomical texture information in the image. These results align with recent research, indicating a close relationship between FDG metabolism levels in primary tumor tissue and CRPM, with mucus components prone to causing PM exhibiting lower FDG uptake, although the AUC value was relatively low in this study [9, 46, 47].

Figure 6 illustrates the inference process for two deep-learning models. Pixels in SMs represent attention in the deep learning model, and hot regions are colored based on attention patterns. The model exhibits more attention to the surrounding area of the tumor for CT images, while the attention in PET images demonstrates significant differences in FDG metabolism.

Several limitations should be acknowledged in this study. Firstly, it is a retrospective and single-center study, with a relatively small sample size and potential bias, which may overestimate the performance of the model. Further studies should be conducted in a different database (e.g., multicentre, larger sample size) in the future. Secondly, the diagnosis of some CRPM patients relied on follow-up due to the inability to undergo surgery or laparoscopic exploration for confirmation, resulting in a lack of a gold standard diagnosis. Thirdly, the study utilized a 2D convolutional neural network, converting three-dimensional lesion information into two dimensions, which may disrupt the spatial topological relationship between different tumor layers, potentially leading to the loss of key information for overall tumor evaluation. Fourthly, the classical radiomics model only extracted features from tumor lesions and did not include the surrounding area of the tumor in this study. Lastly, we observed that the model based on clinical and subjective CT findings demonstrated high sensitivity. In the future, integrating the deep learning model with clinical information may achieve higher diagnostic efficiency.

In conclusion, our results indicate that the Radiomics-boosted deep-learning model surpasses the classical radiomics model, SUVmax model, and clinical and subjective CT finding model. This could potentially assist physicians in making more personalized treatment decisions and follow-up plans for patients.

### Supplementary information

ELECTRONIC SUPPLEMENTARY MATERIAL

## Data Availability

The datasets used or analyzed during the current study are available from the corresponding author upon reasonable request.

## Abbreviations

AUC: Area under the curve
CA19-9: Carbohydrate antigen 19-9
CEA: Carcinoembryonic antigen
95% CI: 95% Confidence interval
CRC: Colorectal cancer
CRPM: Colorectal cancer peritoneal metastasis
CT: Computed tomography
FC: Fully connected
18F-FDG-PET/CT: 18F-fluorodeoxyglucose positron emission tomography/computed tomography
NM: Non-metastasis
NPV: Negative predictive value
PM: Peritoneal metastasis
PPV: Positive predictive value
RFM: Radiomic feature map
ROI: Region of interest
SD: Standard deviation
SM: Significance map
SUVmax: Maximum standardized uptake value
