# Supplementary material for: A radiomics-boosted deep-learning for risk assessment of synchronous peritoneal metastasis in colorectal cancer
Source: Insights Imaging. 2024 Jun 18;15:150. doi: 10.1186/s13244-024-01733-5 (PMC11183032; doi:10.1186/s13244-024-01733-5)
Supplement: Supplementary file 1 — ELECTRONIC SUPPLEMENTARY MATERIAL [file 13244_2024_1733_MOESM1_ESM.pdf]

A radiomics-boosted deep-learning for risk assessment of synchronous peritoneal metastasis in colorectal cancer

ELECTRONIC SUPPLEMENTARY MATERIAL

Supplementary methods

Supplementary A1. Synchronous CRPM status evaluation

Pathological examination results from biopsy or surgical resection confirmed the presence of CRPM. In cases where tissue diagnosis was not feasible, additional clinical and/or imaging follow-up was requested following 18F-Fluorodeoxyglucose Positron Emission Tomography/Computed Tomography (18F-FDG-PET/CT) scan. Lesions were considered malignant based on the following criteria: 1) Confirmation of typical malignant features by multi-modal medical imaging, indicating lesion malignancy; 2) significant progression on follow-up imaging or a substantial post-treatment reduction in tumor size and maximum standardized uptake value (SUVmax). The minimum follow-up duration was 6 months, excluding patients who died or were lost during follow-up. PET/CT scan parameters are provided in Supplementary A2. Subjective CT findings included the infiltration of fat around the tumor (clear or blurry) and the maximum thickness of the tumor layer.

Supplementary A2. 18F-FDG-PET/CT Examinations, image preprocessing, and subjective CT finding evaluation

Before the intravenous injection of 18F-FDG, patients fasted for at least 4 hours and maintained a blood glucose level below 150 mg/dl (Nanjing Jiangyuan Andike Positron Research and Development Co. Ltd., China,

radiochemical purity > 95%). Approximately one hour after receiving 4.07 MBq/kg of 18F-FDG, PET/CT scanning was performed using GE Healthcare Discovery™ 710. The CT image acquisition parameters comprised a tube voltage of 120 kV, tube current ranging from 40-80 mA, slice thickness of 3.75 mm, and a matrix size of 512 × 512. Subsequent to the CT acquisition, based on patient height, PET scanning was done in six to eight beds for 2.5 minutes with a 192x192 matrix. Reconstruction was executed using ordered subset expectation maximization, and data were transferred to the AW workstation for PET/CT image fusion and post-processing utilizing Advance Volume Share 5 (AW 4.6) software.

CT and PET images' pixel values were converted into Hounsfield unit (HU) and SUV. We set the pixel value of CT images as the abdominal window (40, 400) to reduce the interference of fat, bone tissue, and other factors on texture features. PET images were resampled using bilinear interpolation to keep the spatial resolution consistent with CT images. The Z-score method was applied to normalize images[1].

PET/CT imaging results were evaluated by two nuclear medicine physicians who were blinded to the patients' medical records. Any discrepancies were resolved through consensus. The subjective CT finding included the infiltration of fat around the tumor (clear or blurry) and the maximum thickness of the tumor layer.

Supplementary A3. The detailed training parameters of deep learning model

All models were implemented with the PyTorch framework in the Python programming language and were trained using an NVIDIA GeForce GTX 3050 GPU. Throughout the model training process, the Adaptive Moment Estimation (Adam) optimizer was employed to optimize the Cross-entropy classification loss, with a learning rate of 0.0001 and a batch size of 32. To enhance the model's ability to generalize, data augmentation techniques, such as RandomHorizontalFlip and RandomVerticalFlip, were applied.

Supplementary A4. Classical radiomics model construction

For the classical radiomics model, two nuclear medicine physicians independently segmented all volumes of interest (VOI), identifying lesions on PET and CT images based on FDG uptake in colon cancer management. Spherical regions of interest outlined lesions on PET images, generating semi-automated 3D VOIs with a threshold of 2.5. In cases where PET and CT images were well-aligned, the PET-derived VOI was directly transferred to the corresponding CT images. For lesions with low FDG uptake, VOIs were delineated on CT images and mapped onto PET images. To address mismatched lesion borders on PET and CT, separate VOIs were generated for each modality. A second correction, performed by a radiologist with 20 years of experience in medical image interpretation, enhanced the accuracy of the marked tumor area (Fig. S3 a, b, d, e).

Radiomics features were extracted from all segmented CT and PET images using Pyradiomics (http://www.radiomics.io/)[2]. A total of 2104

features, encompassing tumor intensity, shape, size, texture, and wavelet attributes, were extracted from manually delineated regions of interest (ROIs) in primary tumors. The Mann-Whitney-U test and the least absolute shrinkage and selection operator (LASSO) were employed to select the most predictive features in the training dataset (Fig. S3 c, f). Features with p-values greater than 0.05 were discarded as insignificant. Additionally, for fairness, multivariable logistic analysis was employed to establish a classic radiomics model.

Supplementary A5.

$Score_{CT}$ = 0.071470*original_glszm_GrayLevelNonUniformityNormalized + 0.081924*log_sigma_5_mm_3D_glcm_MCC - 0.269224*wavelet_LLH_gldm_DependenceVariance - 0.00397*wavelet_HHH_glcm_SumEntropy

$Score_{PET}$ = 0.306764*wavelet_LLH_firstorder_Median - 0.090634*wavelet_HLH_firstorder_Skewness

$Score_{PET/CT}$ = -9.848522 + 9.613389* $Score_{CT}$ + 8.905102*$Score_{PET}$

1. glszm_GrayLevelNonUniformityNormalized (GLNN):

$$GLNN = \frac{\sum_{i=1}^{N_g}\left(\sum_{j=1}^{N_s} P(i,j)\right)^2}{N_z^2}$$

GLNN measures the variability of gray-level intensity values in the image, with a lower value indicating a greater similarity in intensity values. This is the normalized version of the GLN formula.

2. glcm_ Maximal Correlation Coefficient (MCC):

$$MCC = \sqrt{\text{second largest eigenvalue of Q}}$$

$$Q(i,j) = \sum_{k=0}^{N_g} \frac{p(i,k)p(j,k)}{p_x(i)p_y(k)}$$

The Maximal Correlation Coefficient is a measure of complexity of the texture and 0≤MCC≤1.
In case of a flat region, each GLCM matrix has shape (1, 1), resulting in just 1 eigenvalue. In this case, an arbitrary value of 1 is returned.

3. gldm_DependenceVariance (DV):

$$DV = \sum_{i=1}^{N_g}\sum_{j=1}^{N_d} p(i,j)(j-\mu)^2, \text{ where} \mu = \sum_{i=1}^{N_g}\sum_{j=1}^{N_d} jp(i,j)$$

Measures the variance in dependence size in the image.

4. glcm_SumEntropy:

$$sumentropy = \sum_{k=2}^{2N_g} p_{x+y}(k)\log_2(p_{x+y}(k)+\epsilon)$$

Sum Entropy is a sum of neighborhood intensity value differences.

5. firstorder_Median:
The median gray level intensity within the ROI.

6. firstorder_Skewness:

$$skewness = \frac{\mu_3}{\sigma^3} = \frac{\frac{1}{N_p}\sum_{i=1}^{N_p}(\mathbf{X}(i)-\bar{X})^3}{\left(\sqrt{\frac{1}{N_p}\sum_{i=1}^{N_p}(\mathbf{X}(i)-\bar{X})^2}\right)^3}$$

Skewness measures the asymmetry of the distribution of values about the Mean value. Depending on where the tail is elongated and the mass of the distribution is concentrated, this value can be positive or negative.

1. Zhang Z, Cheng Y, Liu NC (2014) Comparison of the effect of mean-based method and z-score for field normalization of citations at the level of Web of Science subject categories. Scientometrics 101(3):1679-1693
2. van Griethuysen JJM, Fedorov A, Parmar C et al (2017) Computational Radiomics System to Decode the Radiographic Phenotype. Cancer Res 77(21):e104-e107

Fig. S1

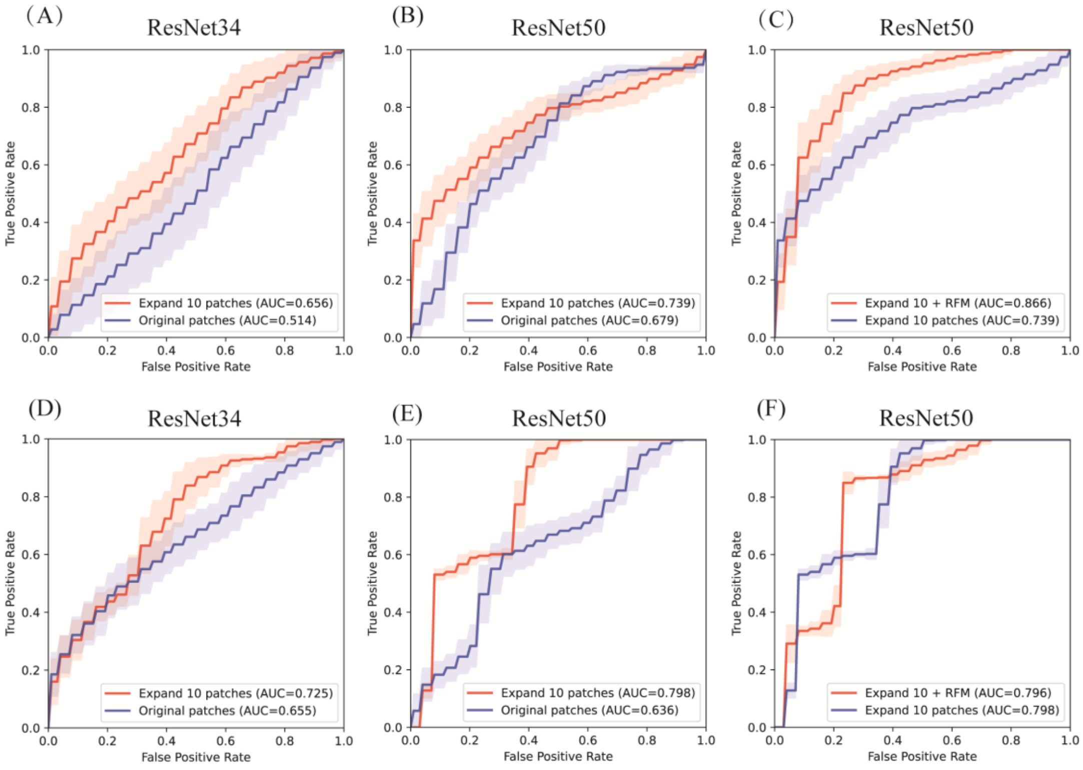

Average AUC of pilot models in internal validation cohort. A-C are based on CT image models, and D-F are based on PET image models. The performance of models based on resnet34 (A and D) are lower than resnet50 (B and E), and the expanded ROI shows better model performance. The performance of CT-based models significantly improved after adding RFM, while PET models slightly decreased (C and F). AUC, area under the curves; ROI, region of interest; RFM, radiomics feature map.

Fig. S2

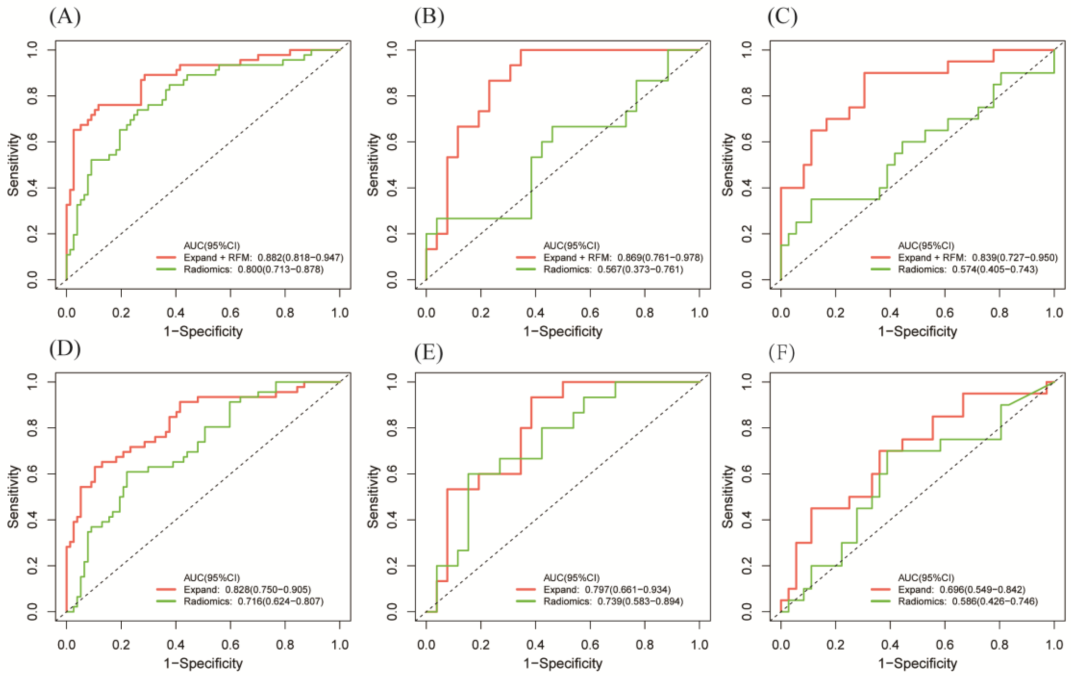

Comparison of classical radiomics and deep learning models based on PET CT images. AUC comparing the CT-based radiomics-boosted deep-learning model and classical radiomics model in the training, internal, and external validation cohorts (A-C). AUC curves comparing the PET-based deep-learning model and classical radiomics model in the training, internal, and external validation cohorts (D-F). AUC, area under the curves.

Fig. S3

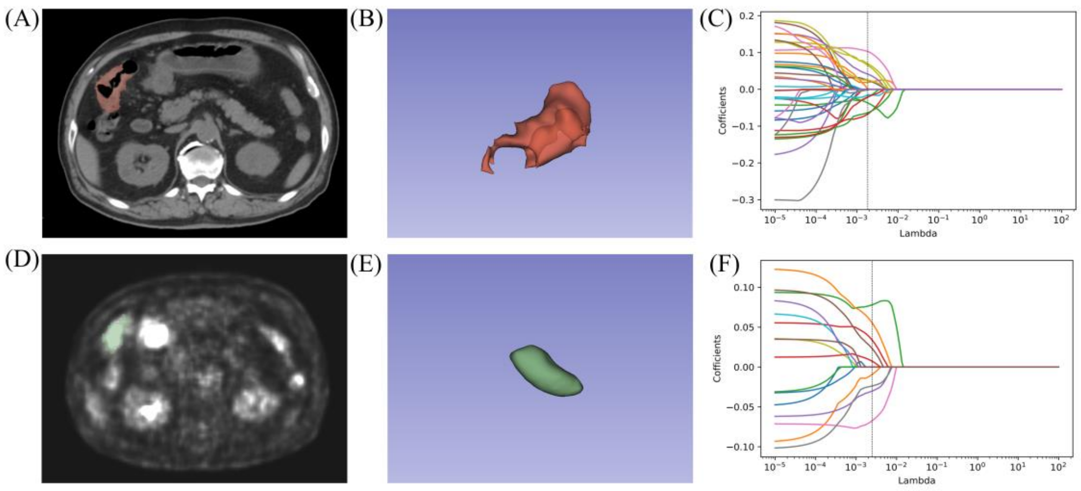

ROI outline and classic radiomics feature screening diagram. ROI generation in CT radiomics (A, B). LASSO analysis for selecting CT radiomics features, with the optimal λ value of 0.0018 identified through 10-fold cross-validation, indicated by the vertical black line (C). Process of ROI generation in PET radiomics (D, E). LASSO analysis for selecting PET radiomics features, with the optimal λ value of 0.0025 identified through 10-fold cross-validation, indicated by the vertical black line (F). ROI, region of interest; The least absolute shrinkage and selection operator.
